# Influence of repeated estrus synchronization treatment on hormone secretion, growth, and development of dairy goats

**DOI:** 10.3389/fvets.2023.1333633

**Published:** 2024-01-10

**Authors:** Shuang Sun, Ming Lv, Huimin Niu, Jun Luo

**Affiliations:** ^1^College of Life Science and Agricultural Engineering, Nanyang Normal University, Nanyang, Henan, China; ^2^Shaanxi Key Laboratory of Molecular Biology for Agriculture, College of Animal Science and Technology, Northwest A&F University, Yangling, Shaanxi, China

**Keywords:** repeated estrus synchronization treatment, hormone antibody, pregnant mare serum gonadotropin, progesterone, dairy goat

## Abstract

In large-scale intensive farms, dairy goats often undergo frequent estrus synchronization (ES) treatment, which may result in a decline in reproductive performance; however, the underlying mechanism remains unclear. The present study aimed to investigate the effect of pregnant mare serum gonadotropin (PMSG) and progesterone (P4)-mediated ES treatment on fertility in dairy goats, while also identifying key metabolic and endocrine mechanisms that influence reproductive performance in does subjected to repeated ES treatment. Forty-eight Saanen does were randomly assigned to two groups (24 goats each) that received ES treatments either thrice fortnightly (3-PMSG) or once (1-PMSG) simultaneously with the third ES treatment of the 3-PMSG group during the breeding season. ES treatment was performed via the intravaginal insertion of a controlled internal drug release (CIDR) device impregnated with 300 mg P4, followed by 300 IU PMSG injections 48 h before CIDR withdrawal. Blood was collected to detect the level of hormones and blood biochemical indices. Additionally, estrus rate, fecundity rate, body weight, size, and lactation performance were measured. The results showed that repeated ES treatment markedly decreased the estrus rate and fecundity rate of goats. Among the does in all groups, there was no substantial difference in follicle stimulating hormone, luteinising hormone, gonadotropin-releasing hormone, melatonin, growth hormone, PMSG, total cholesterol, total protein, and glucose levels, as well as the body weight, body size, and lactation performance. Repeated ES treatment elevated estrogen (E2) levels 36, 48, and 72 h post-CIDR removal; increased P4 upon CIDR insertion; and raised PMSG antibody levels 24, 48, and 72 h post-CIDR removal. The results suggest that elevated anti-PMSG levels are the primary reason for the decline in ES efficiency, and that high E2 and P4 levels at some time points also impair reproductive performance. These findings provide novel insights into the metabolic effects of repeated PMSG stimulation in goats, guiding future reproductive hormone use in breeding practices.

## Introduction

Estrus synchronisation (ES) is a technique to alter the estrus cycle of a group of female animals using exogenous hormones and other methods, causing them to ovulate over the same period (1). The predominant hormones for ES are pregnant mare serum gonadotropin (PMSG) and progesterone (P4) (2). The combination of intramuscular PMSG injection and vaginal P4 suppositories is widely employed to enhance ES efficiency in dairy goats (3, 4).

PMSG is a gonadotropin produced in the chorionic membrane of a pregnant mare, acts as both follicle-stimulating (FSH) and luteinising hormone (LH) (5). PMSG promotes follicular growth, ovulation, and luteinisation in animals (6, 7). P4 can inhibit estrus cycle and arrest follicle development in the luteal phase of cattle (8) and goats (9). Inhibited follicles were stimulated by placing a controlled internal drug release (CIDR) device impregnated with 300 mg of P4 in the goat’s vagina for 10 d (day) (4). Removing CIDR eliminates the effects of P4, prompting the doe to exhibit estrus behavior and ovulate as necessary (10).

Dairy goats often undergo multiple ES treatments throughout their reproductive lifespan to enhance reproductive efficiency and economic benefits (11, 12). Nevertheless, repeated PMSG-mediated ES treatment can seriously impair the reproductive performance (13), even with up to a 1 year interval between each treatment (14). Studies have demonstrated that the repeated PMSG stimulation increases PMSG antibody levels, resulting in PMSG autoimmunity in goats (11, 13). This phenomenon ultimately results in reduced ovarian hormone tolerance (15) and impaired ovary function (7).

Notably, PMSG and CIDR containing P4 are often combined in ES treatment for does (3, 5). However, the impact of vaginal P4 devices during repeated ES treatments on P4 antibody levels and reduced fecundity remains poorly understood. Moreover, gonadotropin-releasing hormone (GnRH), FSH, LH, E2, and P4, which are hormones secreted by the hypothalamic–pituitary-ovary axis, along with growth hormone (GH) and melatonin (MT), play a crucial role in maintaining normal reproductive and physiological functions in animals (16–21). Therefore, comprehensive monitoring of the concentration of main reproductive hormones, PMSG, P4, and their antibodies, is crucial to determine the specific hormone or antibody responsible for reducing reproductive performance caused by repeated ES treatment in does.

Among the key metrics for measuring the economic value of dairy goats, lactation performance is particularly pivotal (22). Yan et al. (23) found that a five-fold repetition of the superovulation treatment in Rhesus monkeys with FSH caused abnormal Golgi apparatus morphology in the mammary follicle tissue and reduced proliferation efficiency of mammary epithelial cells. However, few studies have investigated the changes in the lactation performance of animals that have received repeated ES treatments. In addition, biomarkers of production performance and physical health of goats include body weight and size, and blood biochemical indicators, such as total cholesterol (TC) and protein (TP), and glucose (GLU) (24, 25). However, the effects of repeated ES treatments on reproductive hormones, lactation performance, and blood biochemical and body condition indicators of goats have not been fully explored.

To evaluate the impact of repeated PMSG and P4 mediated ES treatments on production performance and identify the involved endocrine pathways, the present study extensively measured the levels of key hormones (FSH, LH, E2, P4, GnRH, MT, and GH, PMSG) and related antibodies (anti-PMSG and anti-P4), and the estrus rate and fecundity rate of goats. Additionally, this study assessed blood biochemical marker (TC, TP, and GLU) levels, goat milk nutrient composition, and growth indicators in does undergoing ES treatments either once or thrice. The findings aim to establish a theoretical foundation for the judicious use of ES technology in mitigating the adverse side effects caused by repeated hormone stimulation in dairy goats.

## Materials and methods

### Animals

Forty-eight 2.5-year-old Saanen dairy goat were used for the experiment. Each goat had a body condition score of 3, one parity, and one kid from the previous litter, demonstrating good homogeneity. All does were group-housed in yards at the Breeding Station of Xinong Saanen Dairy Goat, Yangling District, Xianyang City, Shaanxi Province, China (Latitude 34°16′N, Longitude 108°4′27.95″E). Throughout the experimental period, the goats had unrestricted access to drinking water and were offered a consistent ration, including 700 g alfalfa hay, 2,800 g silage maize, and 400 g milled concentrate containing corn, soybean meal, rapeseed meal, wheat bran, calcium hydrogen phosphate, salt, and a mix of trace minerals and multivitamins. The study and experimental procedures were approved by the Ethics Committee of Nanyang Normal University under approval number 2021018.

### ES treatment

**Figure 1:**
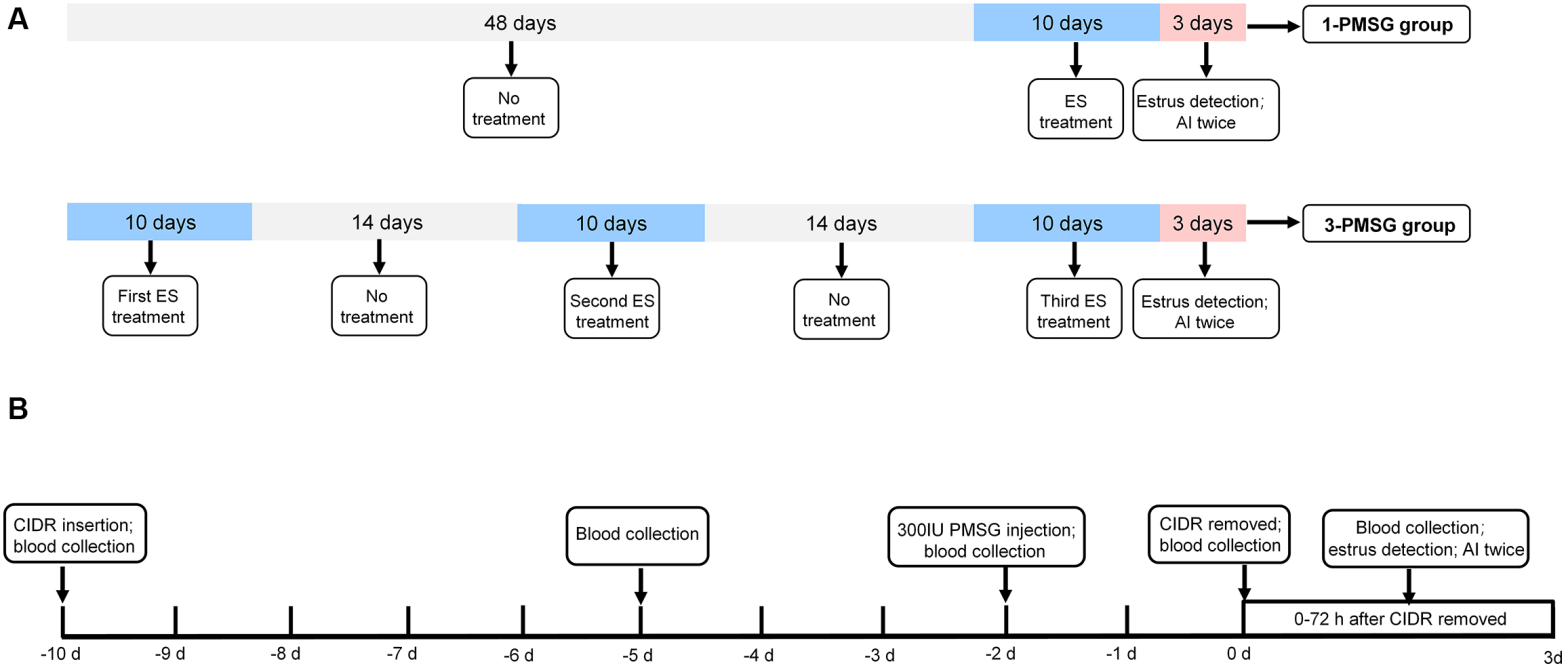
**(A)** The estrus synchronization protocol for 1-PMSG and 3-PMSG group. The ES protocol was achieved by intravaginal insertion of a CIDR device impregnated with 300 mg progesterone, which was retained for 10 d, followed by intramuscular injections of 300 IU PMSG 48 h before the CIDR withdrawal. **(B)** The schedule for 1-PMSG group and 3-PMSG group. In 3-PMSG group, 24 goats were treated with ES three times at 14 d intervals, whereas in 1-PMSG group, 24 goats were treated with ES once concurrently with the third ES protocol for goats in 3-PMSG group. AI, artificial insemination.

As shown in Figure 1A, 48 does were randomly assigned to two groups: the 3-PMSG group, receiving ES treatment thrice fortnightly, and the 1-PMSG group, receiving only once ES treatment, synchronized with the thrice ES treatment of the 3-PMSG group during the breeding season. The interval between ES treatment in the 3-PMSG group was 14 d. Each group contained 24 goats.

Following the protocol from previous research (3), ES treatment in each group involved using a CIDR device impregnated with 300 mg P4 [Pharmacia&Ujohn Company, New York, NY, United States (United States of America)], which was introduced into and maintained in the vagina of each goat for 10 d. Additionally, PMSG was intramuscularly injected in each goat at a dose of 300 IU (Sansheng Corporation, Ningbo, China) 48 h before the withdrawal of the CIDR device (Figure 1B).

One vasectomized buck was used to ensure that the goats were in estrus at 0–72 h after CIDR withdrawal. In addition to the 1-PMSG group, the estrus rate of goats treated with once, twice, thrice ES stimulation in the 3-PMSG group was also calculated. In 3-PMSG group, the estrus goats treated with once, twice ES stimulation were not mated. Subsequently, the estrus goats treated with thrice ES in 3-PMSG group, and estrus goats in 1-PMSG group were both artificially inseminated twice, with a 12 h interval (Figures 1A,B).

### Blood sampling

At 6 a.m. on the first day of ES treatment, blood samples were collected, and the CIDR was inserted into the vagina of each goat in both groups. At 6 a.m. on the fifth day of ES treatment, the blood samples were collected from all does. At 6 a.m. on the ninth day of ES treatment, each dose was intramuscularly injected with 300 IU PMSG. On the tenth day of ES treatment, the CIDR was removed from the vagina of all the goats, and blood samples were also collected. Subsequently, blood samples were collected from all goats at 12 h intervals up to 72 h after CIDR withdrawal, at both 6 a.m. and 6 p.m. Additionally, blood samples were collected from all pregnant goats at 6 a.m., during the first, second, third, and fourth months of gestation and at the time of labour.

At these time points, 2 mL of blood from the jugular veins of does were collected and placed in a 5-mL centrifuge tube containing 200 μL citric acid glucose solution as a plasma anticoagulant. Subsequently, the blood samples were centrifuged at 3,260 g for 15 min at 4°C, using a Sorvall ST8 centrifuge (Thermo Fisher Scientific Technology Co., Ltd., New York, United States).

### Hormonal analysis

Enzyme-linked immunosorbent assay (ELISA) kits (catalogue number: H101-1-2, H206-1-2, Jiancheng Bioengineering Research Institute, Nanjing, China) were used to determine plasma FSH and LH concentrations. The serum levels of E2 and P4 were detected using ELISA kits (catalogue number: TJ0514, TJ0165, Tianjian Biopharmaceutical Co., Ltd., Tianjin, China). Serum level of GnRH, MT, PMSG, GH, anti-PMSG, and anti-P4 was examined using ELISA kits (catalogue number: BYE80207, BYE30198, BYE20197, BYE40026, BYEB20198, BYE11511, Bangyi Biopharmaceutical Co., Ltd., Tianjin, China). Serum concentrations of TC, TP, and GLU were examined using assay kits (catalogue number: BC1985, PC0020, BC2505, Solarbio Science & Technology Co., Ltd., Beijing, China).

### Body size indicators

$$\begin{array}{l} \mathrm{Estrus rate}=\\ \frac{\mathrm{number of estrus goats}}{\mathrm{total number of goats that received}\;ES\;\mathrm{treatment}\left(s\right)}\times 100\%\text{.} \end{array}$$

$$\begin{array}{l} \mathrm{Fecundity rate}=\\ \frac{\mathrm{number of offspring}\;}{\mathrm{total number of goats that received}\;ES\;\mathrm{treatment}\left(s\right)}\times 100\%\text{.} \end{array}$$

The goats were weighed at the time of CIDR insertion and delivery, and their body length, height, and chest and cannon circumferences were measured. The measurement methods of body size indicators were as follows:

Body length: The linear distance from the shoulder to the ischial end of the does.

Body height: The height perpendicular to the ground from the highest point on the dorsal side of the thoracic spine of the does.

Chest circumference: Measurement of the perpendicular line at the posterior edge of the scapula of the does by using a tape measure around the chest cavity.

Cannon circumference: The circumference of the upper 1/3 (thinnest) of the metacarpal bone in the left forelimb of the does.

### Lactation performance

After delivery, 50 mL of milk samples were collected twice at 8:00 a.m. and 6:00 p.m. every 30 d from both 1- and 3-PMSG does during the lactation period. The ratios of milk fat and protein, lactose, total fat solids, and non-fat solids in goat milk, and the density and freezing point of goat milk, were determined using a milk composition analyser (Milkscan FT120 Milk Composition Analyzer; FOSS Group, Copenhagen, Denmark). In addition, the number of lactation days throughout the lactation period after lambing was determined, and the daily milk yield was recorded.

### Statistical analysis

Experimental data were analysed using SPSS 19.0 (SPSS Inc., Chicago, IL, United States). Percentage data were analysed using the chi-square test. For the two groups of data with measurement times greater than or equal to three, the repeatability test was used to analyse and determine the significance of the difference between groups and time and the interaction between these factors. For indicators with less than three measurement times, a homogeneity test was conducted, and either a non-parametric or an independent sample *t*-test was used to analyse the data depending on the data distribution. Data were analysed using a one-way analysis of variance for groups with values greater than or equal to three. The *x^2^* test was used to compare the differences in the estrus rate and fecundity rate of does. The results were then plotted using GraphPad Prism 7 (GraphPad Software, San Jose, CA, United States). All data except percentages are expressed as mean ± standard error. A *p*-value of *<0.05* indicated a significant difference, *p < 0.01* indicated an extremely significant difference.

## Results

### Effects of repeated ES treatment on the estrus rate and fecundity rate of dairy goats

As shown in Table 1, the estrus rate of does treated with once ES stimulation in 3-PMSG group was significantly higher than that of does treated twice, twice ES stimulation in 3-PMSG group (*p <* 0.05), and the estrus rate of 1-PMSG goats was significantly higher that of does treated with once, twice ES of 3-PMSG group. There was no significant difference in oestrus rate between 1-PMSG goats and does treated with the ES treatment once in the 3-PMSG group (*p* > 0.05; Table 1). In addition, the fecundity rate of 3-PMSG goats was extreme significantly lower than that of 1-PMSG goats (*p <* 0.01; Table 2).

**Table 1:** Effects of repeated estrus synchronization (ES) treatment on estrus rate of does in 1-pregnant mare serum gonadotropin (PMSG) and 3-PMSG goats.

Items	1-PMSG group	3-PMSG group		
		Once ES treatment	Twice ES treatment	Trice ES treatment
Number of estrus goats (n)	23	24	11	13
Estrus rate (%)	95.83^a^	100.00^a^	45.83^b^	54.17^b^

a-b indicated within a row with different superscripts differ at *p* < 0.05.

**Table 2:** Effect of repeated ES treatment on fecundity rate of does in 1-PMSG and 3-PMSG group.

Items	1-PMSG group	3-PMSG group	*p* value
Number of kids (*n*)	29	8	—
Fecundity rate (%)	120.83	33.33	< 0.01

### Effects of repeated ES treatment on the concentrations of steroids and gonadotropin hormones in dairy goats

**Figure 2:**
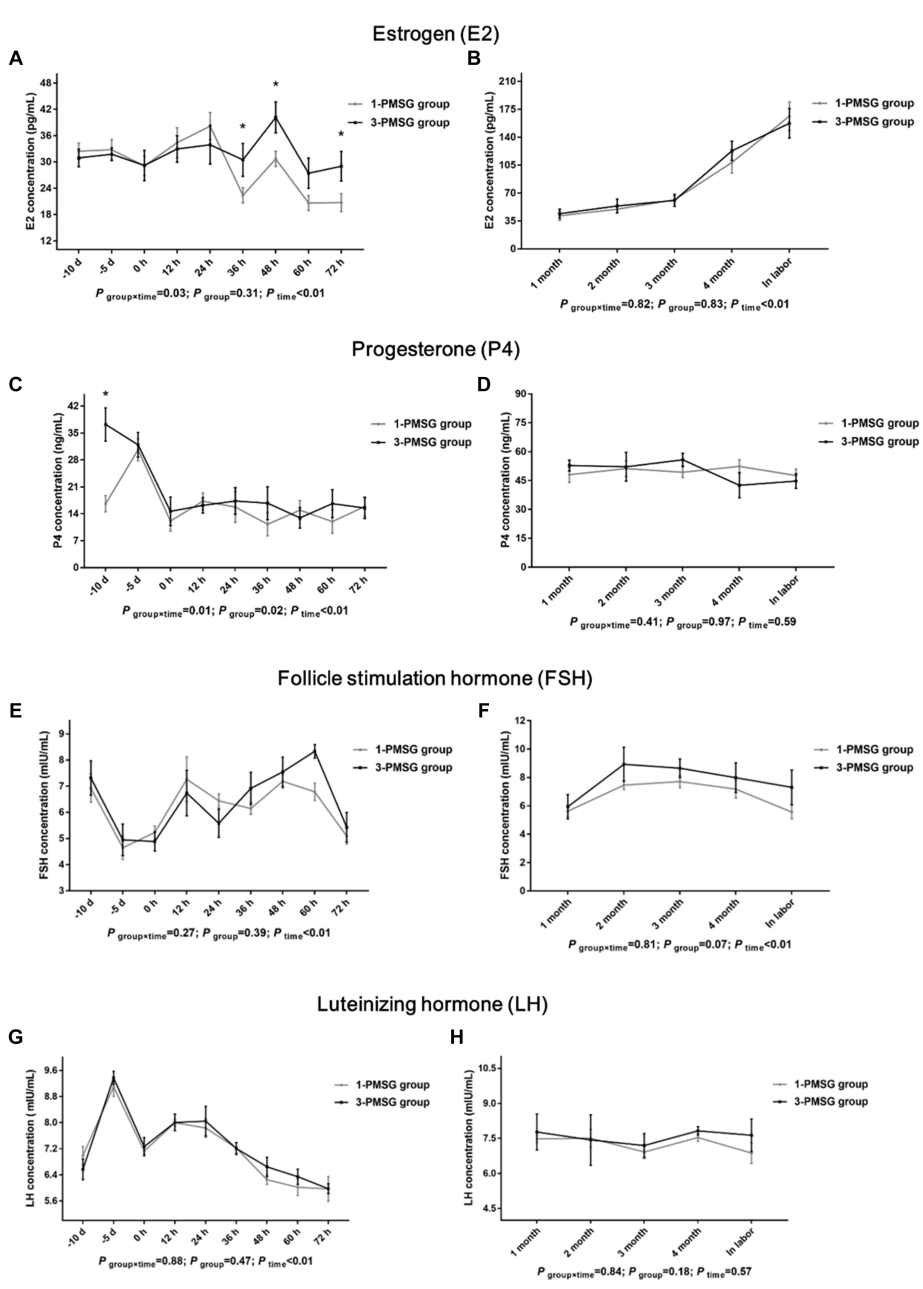
Effects of repeated ES treatment on the concentrations of E2, P4, FSH, and LH in dairy goats. Concentrations of E2 **(A,B)**, P4 **(C,D)**, FSH **(E,F)** and LH **(G,H)** during ES treatment **(A,C,E,G)** and gestation **(B,D,F,H)** period in the goats of 1- and 3-PMSG groups. Legends: “-10 d,” “-5 d,” and “0 d” indicate the day of CIDR insertion, 5 d after CIDR insertion, and the day of CIDR removal, respectively; “0 d, 12 h, 24 h, 36 h, 48 h, 60 h, and 72 h” indicates 0, 12, 24, 36, 48, 60, 72 h after CIDR removal. “1 month, 2 months, 3 months, 4 months, and in labor” indicates the periods of the gestation; “*P*_time×group_” indicates the *p*-value for the interaction between time and group. “*p*_group_” indicates the *p*-value for group effects. “*p*_time_” indicates the *p*-value for the time effects. “*” indicates a significant difference between the two groups at *p* < 0.05 simultaneously.

In 3-PMSG goats, the plasma E2 concentration was significantly higher than that in 1-PMSG goats at 36, 48, and 72 h after CIDR removal (*p* < 0.05), with no significant differences observed at the other time points (*p* > 0.05; Figures 2A,B). Furthermore, the P4 levels in 3-PMSG goats were significantly higher than in 1-PMSG goats at CIDR device insertion (*p* < 0.05; Figure 2C). Throughout pregnancy, both groups maintained elevated P4 levels, with no significant difference between the groups (*p* > 0.05; Figure 2D). Additionally, no significant differences were observed in FSH and LH concentration between the groups at each time point (*p* > 0.05; Figures 2E–H).

**Figure 3:**
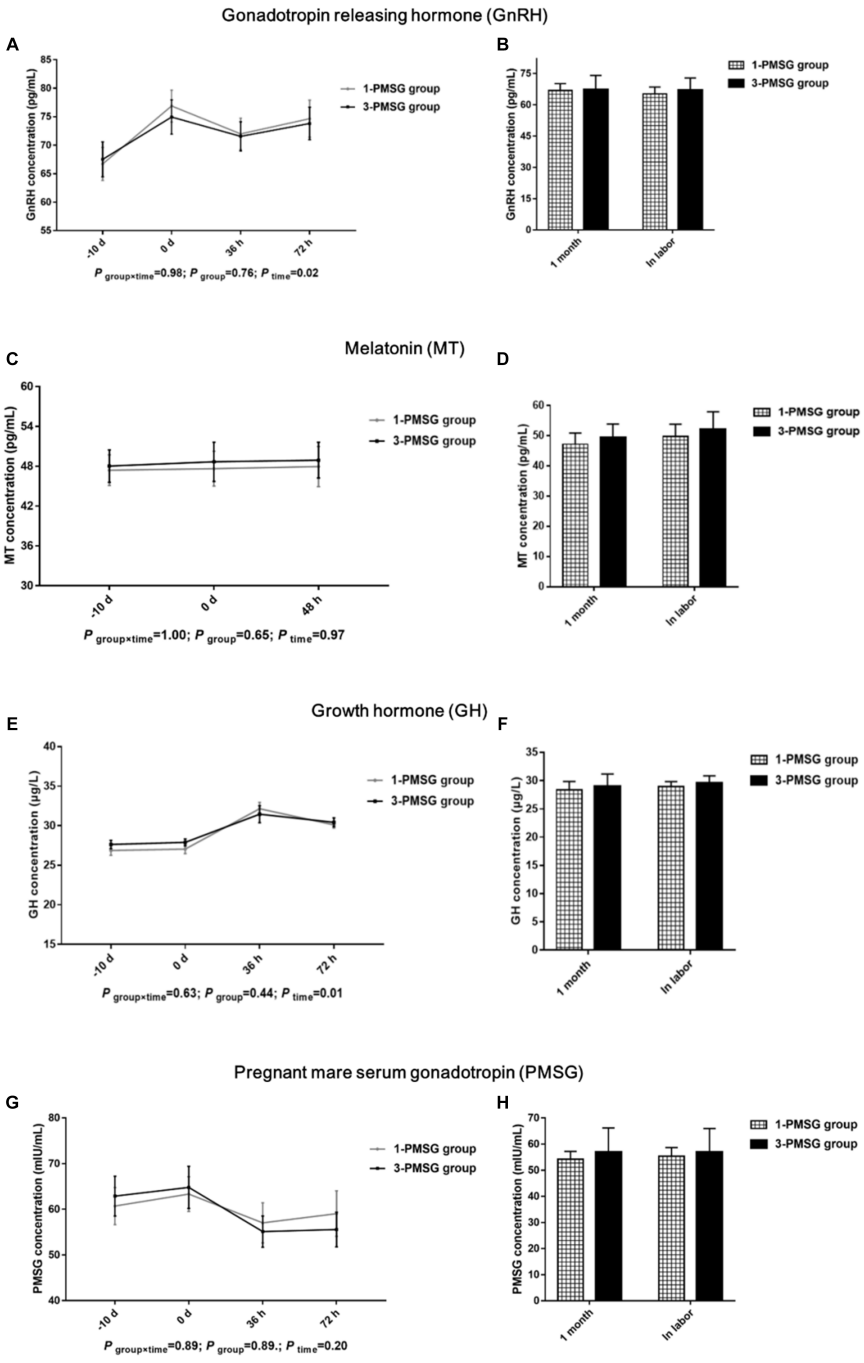
Effects of repeated ES treatment on the concentration of GnRH, MT, GH, and PMSG in dairy goats. Concentrations of GnRH **(A,B)**, MT **(C,D)**, GH **(E,F)** and PMSG **(G,H)** during ES treatment **(A,C,E,G)** and gestation **(B,D,F,H)** period in the goats of 1- and 3-PMSG groups. Legends: “-10 d” and “0 d” indicate the day of CIDR insertion, and the day of CIDR removal. “0 d, 36 h, 48 h, 72 h” indicates 0, 36, 48, 72 h after CIDR removal. “1 month” and “In labor” indicate the first month of pregnancy and the time of labour for 1-PMSG and 3-PMSG goats.

No significant differences in the GnRH, MT, GH, or PMSG concentrations were observed between 1- and 3-PMSG goats at any of the sampled time points (*p* > 0.05; Figures 3A–H).

### Effects of repeated ES treatment on the concentrations of hormone antibodies in dairy goats

**Figure 4:**
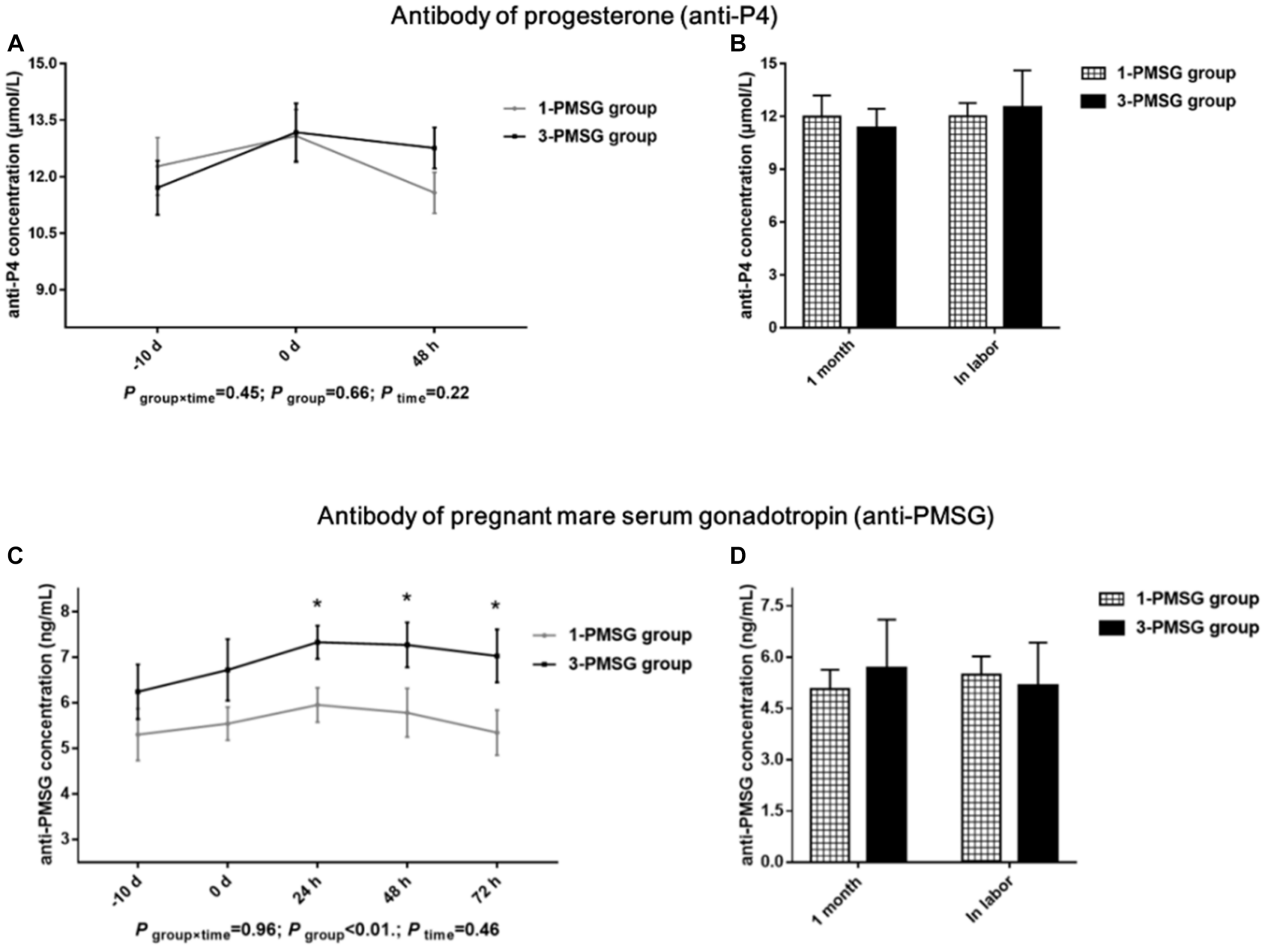
Effects of repeated ES treatment on the concentration of anti-P4 and anti-PMSG in dairy goats. Concentrations of anti-P4 **(A,B)** and anti-PMSG **(C,D)** during ES treatment **(A,C)** and gestation **(B,D)** period in the goats of 1- and 3-PMSG groups.

No significant difference in the anti-P4 concentration was observed between the 1- and 3-PMSG groups (*p >* 0.05; Figures 4A,B). However, the concentration of anti-PMSG in 3-PMSG does was significantly higher than that observed in 1-PMSG at 24, 48, and 72 h after CIDR removal (*p <* 0.05). Conversely, there were no significant differences in anti-PMSG levels between the groups at the time of CIDR insertion or removal (*p >* 0.05; Figure 4C). Furthermore, no significant differences in anti-P4 or anti-PMSG levels were observed between the 1- and 3-PMSG groups during the first month of pregnancy or labour (*p >* 0.05; Figure 4D).

### Effects of repeated ES treatment on the concentrations of blood biochemical indexes and body condition of dairy goats

**Figure 5:**
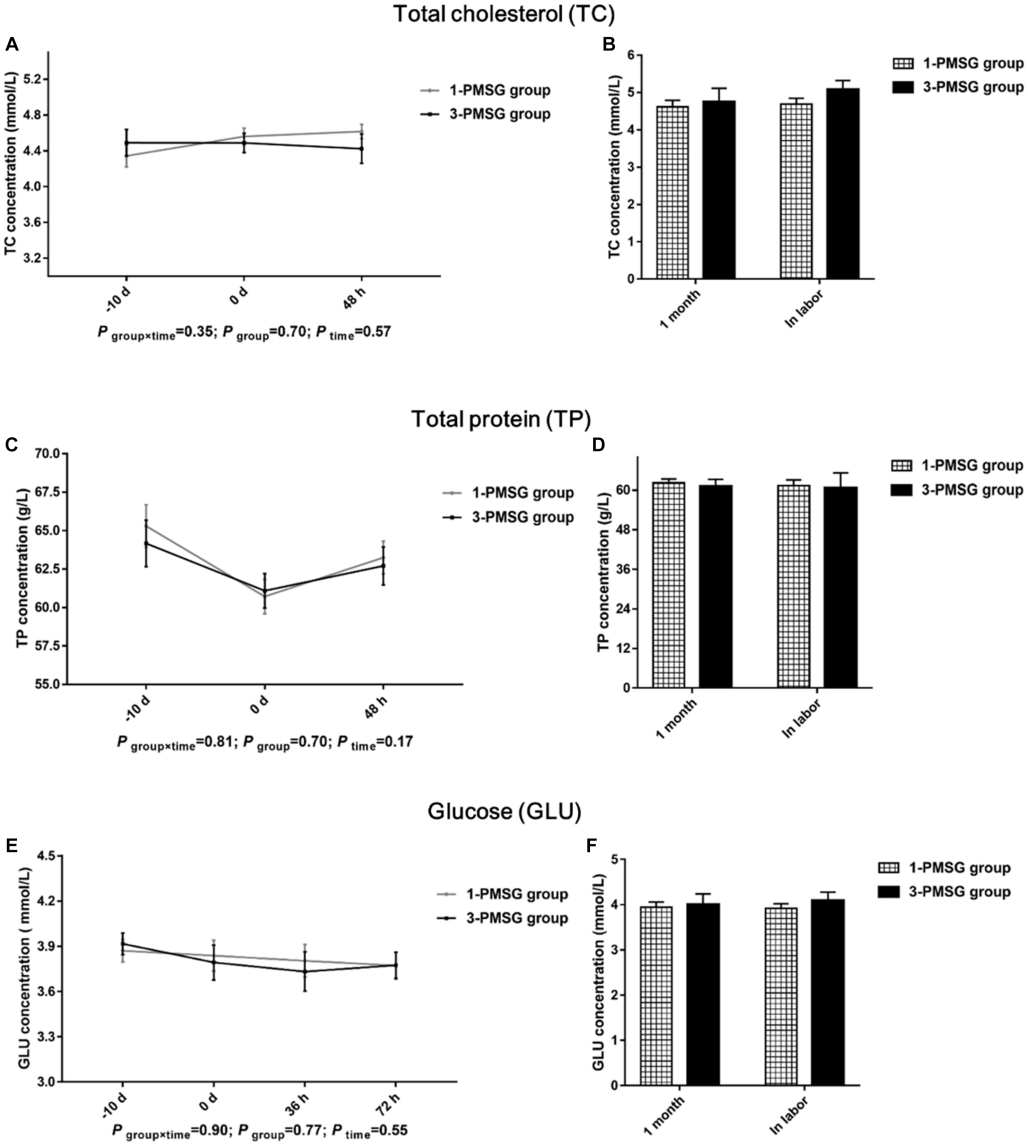
Effects of repeated ES treatment on the concentration of TC, TP, and GLU in dairy goats. Concentrations of TC **(A,B)**, TP **(C,D)** and GLU **(E,F)** during ES treatment **(A,C,E)** and gestation **(B,D,F)** period in the goats of 1- and 3-PMSG groups.

No significant differences were observed in TC, TP, and GLU concentrations between 1- and 3-PMSG does across all sampled time points (*p* > 0.05; Figures 5A–F). Furthermore, there were no significant differences in body weight, length, height, or chest and cannon circumference between 1- and 3-PMSG does at the time of CIDR removal and parturition (Table 3).

**Table 3:** Effects of repeated ES treatment on the weight and body measurements of 1- and 3-PMSG goats.

Items	The day of CIDR removed			The day of does in labor		
	1-PMSG group	3-PMSG group	*p* value	1-PMSG group	3-PMSG group	*p* value
Body weight (kg)	50.64 ± 0.40	50.50 ± 0.40	0.81	56.50 ± 0.15	56.67 ± 0.25	0.56
Body length (cm)	75.62 ± 0.19	76.03 ± 0.22	0.17	76.17 ± 0.30	77.08 ± 0.42	0.11
Body height (cm)	72.84 ± 0.21	72.93 ± 0.30	0.79	73.43 ± 0.33	73.05 ± 0.50	0.54
Chest circumference (cm)	86.04 ± 0.17	85.94 ± 0.24	0.73	87.03 ± 0.28	87.08 ± 0.42	0.93
Cannon circumference (cm)	10.00 ± 0.07	9.89 ± 0.14	0.48	10.63 ± 0.24	10.75 ± 0.25	0.78

The data are presented as mean ± standard error.

### Effects of repeated ES treatment on the lactation performance of dairy goats

No significant differences were found in lactation days or average milk yield between 1- and 3-PMSG goats throughout the lactation period postpartum (*p* > 0.05) (Table 4). Additionally, no significant differences were found in fat, protein, lactose, total fat solids, non-fat solids, density, freezing point, and acidity of goat milk during the lactation period between the groups (*p* > 0.05) (Table 4).

**Table 4:** Effects of repeated ES treatment on the lactation performance of 1- and 3-PMSG goats.

Items	1-PMSG group	3-PMSG group	*p* value
Days in mild (d)	281.13 ± 1.70	280.33 ± 3.02	0.81
Milk yield (kg)	2.96 ± 0.07	2.94 ± 0.06	0.85
Milk fat (%)	3.78 ± 0.20	4.12 ± 0.31	0.36
Milk protein (%)	3.53 ± 0.19	3.26 ± 0.13	0.39
Lactose (%)	4.16 ± 0.09	4.08 ± 0.20	0.13
Total solid (%)	12.56 ± 0.33	13.07 ± 0.42	0.40
Nonfat solid (%)	8.69 ± 0.14	8.88 ± 0.19	0.47
Density (g/cm^3^)	1028.04 ± 0.97	1030.15 ± 0.52	0.20
Freezing temperature (°C)	0.51 ± 0.00	0.51 ± 0.01	0.63
Acidity (°T)	6.93 ± 0.19	7.04 ± 0.13	0.73

The data are presented as mean ± standard error.

## Discussion

The present study demonstrated that repeated PMSG and P4 mediated ES treatment reduce estrus rate and fecundity rate of dairy goats. These results are consistent with those of other studies (13, 14). Repeated PMSG treatment has been reported to decrease reproductive performance in animals, but the metabolic and endocrine effects of repeated PMSG and P4 stimulation had not been studied in detail.

Owing to the prolonged half-life of PMSG (40–125 h), residual PMSG can damage follicle maturation and ovulation (26), induce large anovulatory follicles that continuously secrete E2, which may cause E2 levels in peripheral plasma of mouse (27). In this study, the plasma E2 levels in 3-PMSG does were notably higher than those in 1-PMSG does 36, 48, and 72 h after CIDR removal. Elevated E2 levels were shown to peak before ovulation, inducing estrus behavior in does for mating (28). Excessive E2 during late estrus may inhibit ovulation (27), eventually cause lower fertility of the 3-PMSG goats.

In this study, the does in each group exhibited elevated P4 levels, 5 d after CIDR insertion, indicating the effective implementation of vaginal CIDR treatment and successful induction of the artificial luteal phase. This secretion trend aligned with the biological rule of the luteal phase of the estrus cycle (1, 10). Notably, the P4 concentration in 3-PMSG does was considerably higher than that in 1-PMSG does at CIDR insertion. Abnormally high or low P4 levels can be detrimental to reproductive development and pregnancy maintenance (16). The excessively high P4 levels in 3-PMSG does at CIDR insertion may have contributed to disrupting P4 secretion, highlighting the potential effects of repeated PMSG stimulation on the reproductive system.

FSH plays a key role in regulating both follicle recruitment and atretic follicle development (17, 18). Similarly, LH influences the growth of dominant follicles and oocytes while triggering the apoptosis of preantral follicles (18, 29). Additionally, GH can synergise with FSH and LH, promoting follicle maturation and oocyte excretion (30, 31). Our investigation revealed no substantial differences in plasma levels of FSH, LH, GH, or GnRH between 1- and 3-PMSG does during the estrus cycle and pregnancy periods (*p* > 0.05). These results suggest that repeated PMSG treatment only affect the formation of E2 and P4 secreted by the ovary, not the secretion of hypothalamus and pituitary hormones in dairy goats.

Melatonin is involved in regulating seasonal animal reproduction (20, 32). In an environment with varying light intensity, MT secretion in the body also varied considerably (33). In the present study, no notable differences were observed in MT concentration between groups on the day of CIDR insertion, the day of removal, and 48 h after CIDR removal. This absence of difference might be attributed to the consistent timing of the ES treatment in both groups, suggesting the reliability of our results.

Although vaginal P4 insertion is widely used in ES protocols (8, 10), few reports have elucidated the effects of repeated insertion on P4 antibody expression. This study found no marked differences in levels of the P4 antibody were observed between groups. A high-quality CIDR device was used for the present ES treatment, and rigorous sterilisation and operation standards were used during CIDR device insertion. Thus, a conclusion was that the repeated single use of high-quality CIDR devices under stringent hygiene conditions does not cause an increase in the P4 antibody of does.

PMSG, with physiological activities similar to those of FSH and LH, influences follicle development and hormone secretion and is often used with P4 for the ES of dairy goats (3, 34). Further, anti-PMSG forms a macromolecular antigen–antibody complex *in vivo*, neutralising PMSG and reducing its biological titre (14, 15). This study found that repeated ES treatment with PMSG led to a notable increase in plasma anti-PMSG concentrations at 24, 48, and 72 h post CIDR removal. In agreement with previous studies (13, 14), this study found that the repetition of ES treatment in goats by PMSG was followed by an increase in the antibody of PMSG. Notably, no distinct differences in PMSG levels between groups were observed, suggesting that although a certain level of PMSG may persist due to its long half-life, high anti-PMSG levels in 3-PMSG goats were able to neutralise residual PMSG. The antibodies for PMSG weakened the biological activity of PMSG whilst reducing the efficiency of ES, ultimately reducing the reproductive performance of does (11). Thus, the high anti-PMSG levels explain the reduced reproductive performance of Saanen dairy goats after repeated PMSG treatments.

In cases of impaired health and dysplasia, TC, TP, and GLU contents are affected in humans (35) and goats (25). However, in this study, repeated ES treatments did not influence these blood biochemical indices. Moreover, they did not affect the body weight and size of 1- and 3-PMSG does. Intriguingly, a prior investigation demonstrated adverse effects on mammary gland structure after five repeated PMSG-mediated superovulation treatments (23). Furthermore, no substantial differences in lactation volume and goat milk nutrient composition were found between the groups of this study. The results of lactation performance may be because the hormone dose used for ES treatment was lower than that used for the superovulation treatment, with fewer treatments being used here than those used in previous studies. The results suggest that repeated PMSG- and P4-mediated ES negatively affects the reproductive performance of dairy goats but not impact growth or lactation.

## Conclusion

The repeated use of PMSG and P4 in ES treatment substantially decreased the estrus rate and fecundity rates of Saanen dairy goats. In the 3-PMSG does, elevated E2 levels at 36, 48, and 72 h after CIDR removal; increased P4 levels at CIDR insertion; and high anti-PMSG content at 24, 48, and 72 h after CIDR removal were observed. Concentrations of FSH, LH, GnRH, MT, GH, TC, TP, GLU; body condition indicators; and lactation performance were unaffected by repeated ES treatment. The results suggest that elevated anti-PMSG levels are the primary factor contributing to reduced reproductive performance, and elevated E2 and P4 levels can also diminish ES treatment efficiency. These findings provide insights into the endocrine mechanisms influencing fertility after repeated ES treatment, offering a foundation for optimising future applications of ES technology.

## Data availability statement

The raw data supporting the conclusions of this article will be made available by the authors, without undue reservation.

## Ethics statement

The animal study was approved by the Ethics Committee of Nanyang Normal University. The study was conducted in accordance with the local legislation and institutional requirements.

## Author contributions

SS: Formal analysis, Funding acquisition, Writing – original draft. ML: Formal analysis, Methodology, Writing – original draft. HN: Investigation, Project administration, Writing – original draft. JL: Funding acquisition, Supervision, Writing – review & editing.
